# Coagulation in chronic liver disease and the use of prothrombin complex concentrate for an emergent procedure: a case report and review of literature

**DOI:** 10.1080/20009666.2018.1466600

**Published:** 2018-06-12

**Authors:** Matthew Laubham, Eric Kallwitz

**Affiliations:** Department of Hepatology & Department of Internal Medicine, Loyola University Medical Center, Maywood, IL, USA

**Keywords:** 4-factor prothrombin complex concentrate (4F-PCC) (Kcentra), prothrombin time/ international normalized ratio (INR), cirrhosis, coagulopathy

## Abstract

Synthetic dysfunction observed in cirrhosis results in altered production of procoagulants and anticoagulants that can lead to both bleeding and thrombotic events, respectively. In patients with decompensated cirrhosis, frequent hospital visits often require bedside procedures such as diagnostic paracenteses, thoracenteses and endoscopy. It can be difficult to determine at what coagulation threshold procedures can safely be performed. Currently, the most common therapies given pre-procedurally include fresh frozen plasma (FFP) and vitamin K. The effectiveness of these treatments is estimated by international normalized ratio (INR), an imprecise measure of coagulation in the setting of cirrhosis. Transfusion with FFP may lead to detrimental side effects, including worsening volume overload and increased portal hypertension. We present a case of a 60-year-old patient intubated for acute hypoxic respiratory failure secondary to volume overload who subsequently developed bilateral pneumothoraces, requiring immediate chest tube placement. In this case, the patient had ongoing hepatic decompensation with volume overload and acute worsening of coagulopathy with an INR of 4.2. In this setting, 4-Factor Prothrombin Complex Concentrate (4F-PCC) was chosen to correct coagulation parameters with a low infusion volume. One hour following administration, INR was 1.5. Chest tubes were placed bilaterally and oxygenation improved without bleeding complications. While the data is still lacking, 4F-PCC may be considered for urgent and emergency situations in cirrhotic patients.

## 1. Introduction

A 60-year-old male with nonalcoholic steatohepatitis-induced cirrhosis complicated by portal hypertension and esophageal varices presented to the hospital for shortness of breath and respiratory distress. He had multiple prior hospitalizations and was undergoing evaluation for liver transplant. His MELD score was 19 on admission. There was a concern for pneumonia on presentation and he was started on empiric reatment with broad-spectrum antibiotics. On hospital day four, he experienced worsening respiratory distress and developed hypoxic respiratory failure. This occurred despite optimal use of diuresis, noninvasive ventilation and paracenteses. He was intubated on hospital day five. On the day of intubation, his total bilirubin was 2.1, serum creatinine 1.65, international normalized ratio (INR) 2.4 and serum sodium of 142 (MELD of 16). Mechanical ventilation was with assist control with tidal volumes of 350 mL, rate of 32, PEEP of 16 on 90% FiO2. Arterial blood gas showed a pH of 7.25, CO_2_ of 55, and O_2_ of 116.

After intubation, he had progressive and refractory hypoxemia requiring increasing PEEP with high FiO2 to maintain oxygenation. Four days after intubation, he was found to have large apical bilateral pneumothoraces that required emergent bilateral chest tube placement. The development of bilateral pneumothoraces was in part due to increasing PEEP to meet the oxygenation demands. He had progressive coagulopathy during his ICU course as his INR was 4.2 and PT 46.8. It was felt that urgent correction of coagulopathy was required prior to the procedure, and that it should be done without excessive volume administered. Therefore, he was given 2.9 million units of 4-factor prothrombin complex concentrate (4F-PCC) (based on weight) and within 1 h following administration his INR was measured at 1.5. Subsequently, bilateral chest tubes were placed. There was an immediate improvement of oxygenation, and there were no bleeding complications. One day after the placement of the bilateral chest tubes the patient died from ongoing hepatic decompensation.

## 2. Discussion

Clinicians caring for cirrhotic patients are often guided by inaccurate laboratory measures of hemostasis. In patients with cirrhosis, it is common practice to correct coagulation parameters prior to a procedure; however, this practice is not based on data or guidelines. Numerous studies have found that bleeding after procedures is not predicted by routinely measured coagulation parameters [1–3]. Guidelines from the American Association for the Study of Liver Disease (AASLD) do not recommend routine correction of coagulopathy prior to elective paracenteses [4]. Additional data suggests such cirrhotic patients can safely undergo thoracentesis in the setting of coagulopathy, including a recent retrospective series of 66 cirrhotic patients undergoing thoracentesis that found no major bleeding events irrespective of coagulation parameters or whether coagulation products were administered [5]. Another study included over 1000 patients with coagulopathy (INR greater than 1.6 or platelets less than 50,000) from any etiology that was not corrected prior to thoracentesis found only four bleeding complications among the 303 performed [6]. Although literature suggests that coagulation products do not need to be administered prior to elective procedures in stable patients, less in known about unstable patients with ongoing decompensation and there are no current guidelines addressing this situation.

A significant issue in patients with cirrhosis is how to measure the risk of bleeding, especially as it pertains to a procedure. The INR is the most commonly used measure of coagulation in cirrhosis; however, the INR does not truly reflect coagulation in cirrhotic patients [7]. In fact, the INR was devised and validated for patients on a vitamin K antagonist [8]. Despite this, the INR is used often in clinical practice for cirrhotic patients, elucidating a difficulty in shifting away from this paradigm.

In cirrhosis, there is a disruption of the production of procoagulant (factors II, VII, IX, X) and anti-hemostatic factors (Protein C/S, anti-thrombin) [9]. Therefore, thrombin generation may be altered depending on the relative presence of these procoagulant and anticoagulant factors, as concluded in a study showing thrombin generation in patients undergoing liver transplantation is equal or even superior to thrombin generation in healthy volunteers [10]. For persons taking vitamin K antagonists, the INR is calibrated accordingly [11]. The ambiguity in measuring alterations in clotting factors is affected more by chronic liver disease than by vitamin K antagonism [12]. One study found that when analyzed at different laboratories, the use of different PT reagents and laboratory equipment at different laboratories was found to account for a 26% difference in INR values from the same individuals with cirrhosis [13]. Given a tenuous balance of a disrupted procoagulatant and anticoagulant factor synthesis in liver disease, increasing INR values may reflect worsening synthetic function of the liver, but does not necessarily reflect a higher propensity for bleeding events and only reflects the procoagulant side of this balanced equation [14]. Lowering or normalizing INR values in patients with bleeding events, such as variceal bleeding, does not lead to improved outcomes or decreased failure rates in hemorrhage control [15]. Additionally, a study by Ewe found that INR was not predictive of liver bleeding time after laparoscopic liver biopsy [16].

In regards to the paradigm of patients with liver disease being at a heightened risk of bleeding, several studies refute this concept by identifying significant development of venous thrombosis in these patients [17,18]. In the same shift of concept, liver transplant patients were paradoxically found to have a procoagulant state in the setting of an elevated INR, as well as lower rates of portal vein thrombosis and improved survival without the development of hemorrhagic complications in patients with decompensated liver disease treated with enoxaparin [19,20].

Thromboelastography (TEG), also known as rotational thromboelastography (ROTEG) or rotational thromboelastometry (ROTEM), represents a blood test that may be additive to guide clinical management in cirrhotic patients. TEG studies can be performed as point of care tests. A TEG study provides a more comprehensive coagulation assessment compared to INR and platelet count. This test includes rotating sample blood gently to activate coagulation. Measured parameters include the speed and strength of clotting. TEG measures plasma coagulation, platelet function and fibrinolysis. Data already supports the use of TEG as an effective guide to transfusion during liver transplants [21]. A randomized trial of TEG guided transfusion to standard of care correction in patients undergoing an invasive procedure found a decrease in the transfusion of blood products without an increase in complications [22]. However, little data exist comparing TEG guided transfusion of blood products to no transfusion. A major limitation of TEG is that, despite its bedside capabilities, it is not available in all hospitals or clinical settings.

A final consideration is the routine correction of coagulopathy may have adverse effects. Transfusions may lead to worsening portal hypertension, which is a major contributor to bleeding events in cirrhotic patients. The risk of esophageal and gastric variceal bleeding increases with rising portal pressures. Varices are present in up to 30–40% of compensated cirrhosis at the time of diagnosis, and are present in 85% of Child–Pugh C patients [23,24]. Variceal bleeding in cirrhosis carries a mortality of 7–15% [25]. The use of fresh frozen plasma (FFP) in patients with cirrhosis and portal hypertension has been shown to increase portal pressures in a linear fashion based upon volume [26]. A study by Youssef points out that increased doses of FFP are often required to improve coagulopathy in chronic liver disease patients who were unresponsive to vitamin K therapy, highlighting that six units were associated with greater correction than compared to those receiving less than six units – a significant amount of volume [27]. Another risk and complication of transfusions are transfusion reactions, including transfusion-associated cardiac overload, allergic reactions and febrile transfusion reactions. A study evaluating transfusions in upper gastrointestinal bleeding found a higher rate of transfusion reactions and cardiac overload in the group with unrestricted transfusion [28].

In the current case, a patient was encountered with ongoing decompensation and worsening coagulopathy. The patient required large bore chest tubes and it was decided to reverse coagulopathy in a rapid fashion with a product that would require a low volume to infuse. For these reasons, 4F-PCC was chosen. 4F-PCC is a medication made up of blood clotting factors II, IX and X, and it is FDA approved for urgent reversal of acquired coagulation factor deficiency induced by vitamin K antagonist therapy in adult patients needing an urgent surgery, other invasive procedure, or acute major bleeding. The role of 4F-PCC in patients with coagulopathy from chronic liver disease is not well defined. For reversal of vitamin K antagonist-associated coagulopathy and acute bleeding, 4F-PCC was found to be non-inferior in efficacy compared to FFP with less volume transfused [29]. The 2012 American College of Chest Physicians guidelines recommend 4F-PCC with vitamin K as an alternative to FFP in vitamin K antagonist-associated bleeding because of decreased complications including less volume [30].

The use of 4F-PCC in patients with cirrhosis has been largely limited to case series and expert opinions [31,32]. Studies such as the PROTON trial are currently investigating PCC use in chronic liver disease [33]. One study found PCC was able to lower median INR to 1.3, but the design of the trial allowed no assessment as the whether it reduced bleeding [34]. However, other studies found 4F-PCC to have limited effect in controlling bleeding in cirrhotic patients [35]. The reporting of thromboembolisms as an adverse effect of 4F-PCC in the general population have also caused hesitancy for 4F-PCC use [36,37].

In conclusion, physicians can be sceptical of the need to give coagulation products for stable patients undergoing routine procedures. However, emergent procedures in unstable cirrhotic patients may have a need for coagulation products that is poorly defined, highlighted by this case in which a non-bleeding cirrhotic patient had pre-procedural reversal of an elevated INR. Physicians should be aware of the limitations of simple measures of coagulation in cirrhotic patients such as the INR value. In addition, physicians should be aware of the potential adverse events that can be precipitated by judiciously transfusing coagulation products. Newer agents such as 4F-PCC are being studied in patients with cirrhosis. However, the use of these agents at this time should be limited to clinical studies or other emergent situations until further data is available.
